# The Swiss OMICS‐AD multimodal biomarker cohort study: design, methods, and cohort characteristics

**DOI:** 10.1002/alz70856_106465

**Published:** 2026-01-12

**Authors:** Miriam Rabl, Leonardo Zullo, Jelena Wehrli, Pyry Helkkula, Karolin Hössel, Julius Popp

**Affiliations:** ^1^ Psychiatric University Hospital Zurich and University of Zurich, Zurich, Zurich, Switzerland; ^2^ Department of Psychiatry, Old Age Psychiatry Service, Lausanne University Hospital, Lausanne, Switzerland

## Abstract

**Background:**

Besides amyloid pathology and tau‐related neurodegeneration, multiple other molecular alterations and pathway dysregulations have been observed in Alzheimer's disease (AD). Omics and multi‐omics approaches may help to better understand this complex pathophysiology of AD, and to discover new biomarkers and targets for interventions. The OMICS‐AD study's main scope is to use multi‐omics data to a) better understand pathophysiological changes of AD and related neuropsychiatric symptoms (NPS), and to b) identify and validate new biomarker candidates for AD and AD‐related outcomes such as cognitive decline or persistent NPS. Here, we describe the study design and participant characteristics.

**Method:**

Participants with normal cognition (NC), subjective cognitive decline (SCD), mild cognitive impairment (MCI), and mild AD dementia were included at four Swiss memory clinic centres. Comprehensive clinical and neuropsychological data was collected using validated and widely used neuropsychological tests and questionnaires at baseline, and at follow‐up visits at 18 and 36 months from baseline. Paired blood and CSF samples as well as structural MRI data were obtained at baseline AD status was determined according to CSF AD biomarkers of the core AD pathology. Comprehensive omics analysis will be performed and integrated in multi‐omics data analysis to achieve the main scientific aims of the study.

**Result:**

A total of 434 participants (217 in Lausanne‐1, 138 in Zurich, 56 in Lausanne‐2, and 23 in Bern) were included by the end of 2024. Of these, 200 (46.1%) were cognitively unimpaired (120 were NC and 80 had SCD) and 234 (53.9%) were cognitively impaired (of which 203 had MCI and 31 mild AD dementia). Of the included subjects 50.0% experienced NPS as assessed by the Neuropsychiatric Inventory Questionnaire, the most common symptoms being irritability (19%) and depression (18%). A CSF biomarker profile indicating AD pathology was present in 118 (31.4%) of all subjects (11.5% in NC, 10.3% in SCD, 45.1% in MCI, and 69.0% in mild AD dementia).

**Conclusion:**

By using this large, multicenter, longitudinal, and well‐characterized brain aging cohort we expect to advance the understanding of the pathomechanisms of AD and AD‐related symptoms, while also enabling the identification of novel diagnostic and prognostic biomarkers.

